# Comprehensive genome-wide identification and functional analysis of the GT8 gene family in *Eucalyptus Grandis*

**DOI:** 10.3389/fpls.2025.1610059

**Published:** 2025-06-19

**Authors:** Yufan Jie, Liwan Liu, Linsi Li, Ai-Min Wu, Chunjie Fan, Siwen Liu

**Affiliations:** ^1^ Guangdong Key Laboratory for Innovative Development and Utilization of Forest Plant Germplasm, College of Forestry and Landscape Architectures, South China Agricultural University, Guangzhou, China; ^2^ State Key Laboratory of Tree Genetics and Breeding, Key Laboratory of State Forestry and Grassland Administration on Tropical Forestry, Research Institute of Tropical Forestry, Chinese Academy of Forestry, Guangzhou, China; ^3^ Guangdong Provincial Key Laboratory of Utilization and Conservation of Food and Medicinal Resources in Northern Region, Shaoguan University, Shaoguan, China

**Keywords:** glycosyltransferase, GT8, gene family, *Eucalyptus grandis*, functional analysis

## Abstract

**Introduction:**

The Glycosyltransferase 8 (GT8) family is critically involved in plant cell wall synthesis, yet exhibits significant functional divergence among its members. Despite its importance, systematic characterization of GT8 genes in woody plants remains limited. This study aims to comprehensively analyze the GT8 gene family in Eucalyptus grandis to elucidate its role in cell wall biosynthesis.

**Methods:**

We employed bioinformatics tools to mine the E. grandis whole-genome database. A systematic analysis was conducted, including phylogenetic classification, assessment of physicochemical properties, subcellular localization prediction, gene structure annotation, chromosome mapping, and cis-acting element identification in promoter regions.

**Results:**

Fifty-two GT8 family members were identified and classified into four subfamilies: GAUT, GATL, GolS, and PGSIP. Protein molecular weights ranged from 15.75 to 185.00 kD (mean: 49.08 kD). Genes were dispersed across all chromosomes except chromosomes 3 and 7. Promoter analysis revealed ubiquitous hormone-responsive cis-elements and prevalent light-responsive elements. Phylogenetic inference suggested that EgGUX02 and EgGUX04 may mediate glucuronic acid (GlcA) incorporation into xylan side chains, while EgGAUT1 and EgGAUT12 are likely direct contributors to xylan and pectin biosynthesis.

**Discussion:**

This study provides the first genome-wide functional annotation of the GT8 family in E. grandis, revealing subfamily-specific roles in cell wall polymer synthesis. The enrichment of stress- and hormone-responsive promoter elements implies regulatory complexity in cell wall remodeling. Our findings establish a foundation for targeted manipulation of xylan and pectin biosynthesis in woody plants, with potential applications in biomass engineering.

## 1 Introduction

The cell wall is a unique external protective structure in plant cells, providing mechanical support, maintaining normal metabolic functions, and facilitating responses to environmental stress. It is essential for plant growth and development (Gigli-Bisceglia et al., 2020) As such, the cell wall is closely connected to physiological processes such as absorption, transpiration, transport, and secretion. Plant cell walls are typically comprised of the middle lamella, primary wall, and secondary wall, with primary components including cellulose, hemicellulose, lignin, and pectin (Kubicek et al., 2014).

Pectin, cellulose, and hemicellulose make up the majority of the primary wall. Cellulose is interconnected by hydrogen bonds, providing structural strength and support to the cell wall. Xylan, the predominant hemicellulose in plant cell walls, consists of a β- (1→4)-linked D-xylose backbone with L-arabinose and 4-O-methyl-glucuronic acid side chains (Zabotina, 2012). It mediates interactions between the pectin/lignin matrix and cellulose microfibrils, affecting wall flexibility biomass recalcitrance (Kang et al., 2019; Zhong et al., 2019; Yu et al., 2022). The three types of pectin-homogalacturonan (HG), rhamnogalacturonan I (RG-I), and rhamnogalacturonan II (RG-II)-are structurally complex polysaccharides found in plant cell walls made up of α-D-galacturonic acid residues connected by 1,4-bonds. The most prevalent sugar residue in all plant primary walls is galacturonic acid (GalA) (Ridley et al., 2001). In plant cell walls, HG, a polymer of α-1,4-linked D-galacturonic acid, makes up more than 60% of the pectin (Caffall and Mohnen, 2009). Pectin regulates the cell wall’s porosity, contributing to its plasticity and permeability (Mohnen, 2008). These carbohydrates play critical roles in the composition and metabolism of plant cell walls.

Glycosyltransferases (GTs) are enzymes that transfer sugar moieties from active donor molecules to specific acceptor molecules, thereby catalyzing the creation of glycosidic connections. The manufacture of disaccharides, oligosaccharides, and polysaccharides is facilitated by these enzymes (Sinnott, 1990; Lairson et al., 2008). Glycosyltransferases were categorized into 138 families as of March 2025 based on sequence similarity and substrate recognition (http://www.cazy.org/GlycosylTransferases.html). Among these, the GT8 family has a vital part in plant cell wall production and abiotic stress response (Wang et al., 2024). Evolutionary analysis has classified GT8 into seven subfamilies: Galacturonic acid transferases (*GAUT*), Galacturonic acid transferase-like transferases (*GATL*), GATL-related (*GATR*), Galactinol synthases (*GolS*), and Plant glycogen starch synthesis initiation protein A (*PGSIP-A*), *PGSIP-B*, and *PGSIP-C* (Yin et al., 2010; Kong et al., 2019). The production of pectin and xylan is the main function of GAUT, GATL, and GATR, which also aid in cell wall remodeling (Sterling et al., 2006). PGSIP proteins are involved in initiating starch biosynthesis (Chatterjee et al., 2005), while GolS is a key enzyme in the synthesis of cotton oligosaccharides, playing a role in plant abiotic stress responses (Taji et al., 2002). In *Arabidopsis thaliana*, the GT8 gene family comprises 41 proteins categorized into four major subfamilies: GAUT, GATL, GolS, and PGSIP (Yin et al., 2010). Among these, *QUASIMODO1 (QUA1*) and *GALACTURONOSYL TRANSFERASE1* (*GAUT1*) have been shown to be involved in pectin biosynthesis (Bouton et al., 2002). In particular, *QUA1* is expressed in vascular tissues and influences the activity of β-1→4-D-xylan synthase and α-1→4-D-galacturonic acid transferase, which are involved in the synthesis of pectin and hemicellulose (Orfila et al., 2005). GAUT1 encodes a galacturonic acid polysaccharide glycosyltransferase directly involved in pectin synthesis, and alterations in its function significantly affect plant cell wall composition and function (Caffall and Mohnen, 2009). *AtGATL1/PARVUS* was initially thought to play a role in pectin synthesis (Bouton et al., 2002), However, further studies revealed that the *PARVUS* gene, which is essential for xylan biosynthesis, is expressed in cells undergoing secondary wall thickening. Its loss of function leads to reduced mechanical strength of the plant cell wall (Lee et al., 2007). *AtGAUT12/IRX8 (IRREGULAR XYLEM 8)* is also crucial for xylan synthesis. Both *PARVUS* and *IRX8* contribute to the synthesis of tetramer-saccharides at xylan’s reducing end (Brown et al., 2007; Peña et al., 2007), and *A. thaliana irx8* mutants exhibit reduced xylan and lignin content, along with partial reductions in pectin and cellulose (Persson et al., 2007). Additionally, *GUX1*and *GUX2* are involved in xylan side chain synthesis, particularly by adding GlcA to the xylan backbone (Rennie et al., 2012). In *Populus deltoides*, *PdGATL1.1* and *PdGATL1.2* are the closest orthologs of the *A. thaliana* gene *PARVUS* and function in xylan biosynthesis (Suo et al., 2022). RNAi-mediated knockdown of *GAUT12.1* in *Populus* reduced xylan and pectin content during wood formation, along with decreased biomass recalcitrance. Given the conserved role of *GAUT12* in *A. thaliana*, these results suggest that *GAUT12.1* similarly plays a key role in pectin and xylan biosynthesis in *Populus* (Biswal et al., 2015; Biswal et al., 2018). These genes are vital for plant cell wall construction and mechanical support. For a deeper comprehension of the dynamic process of woody plant cell wall formation, it is imperative to investigate the functional partitioning and evolution of the GT8 gene family.*Eucalyptus* (*Eucalyptus robusta Smith*), a species from the Myrtaceae family, is known for rapid growth, strong adaptability, high economic value and diverse uses. It is one of the three major fast-growing tree species in artificial afforestation, alongside poplar and pine (Kirch et al., 2011). With a short growth cycle, rapid regeneration, and strong soil nutrient dependency, *Eucalyptus* is a key species in southern artificial forests and a critical species for timber reserves (Melesse and Zewotir, 2017). The GT8 glycosyltransferase family has been studied in various species. GT8 family member numbers vary across species: 41 in *A. thaliana* (Yin et al., 2010), 40 in *Oryza sativa* (Kong et al., 2019), 40 in *Solanum lycopersicum* and 56 in *Malus domestica* members (Wang et al., 2024). However, research on the GT8 family in *E. grandis* remains limited. This study identifies the GT8 gene family in *E. grandis* based on bioinformatics analysis. We analyzed various features including physicochemical properties, phylogenetic trees, gene structures, conserved motifs, chromosome locations, and synteny relationships using multiple bioinformatics tools: TBtools-II (v1.120), MEGA11 (v13.0), and Jalview (v2.11.2.6), along with other online platforms. These analyses provide a foundation for further exploring the functional roles of GT8 genes in *E. grandis*.

## 2 Materials and methods

### 2.1 Gene family member identification and physicochemical property analysis

Gene sequences for the GT8 gene family members from *E. grandis* (Myburg et al., 2014) and *A. thaliana* were retrieved from the Phytozome database (https://phytozome-next.jgi.doe.gov/) and the TAIR database (http://www.arabidopsis.org) (Berardini et al., 2015), respectively. The genomic sequences were then converted to protein sequences using TBtools-II (Chen et al., 2023). Comparative alignment of *E. grandis* and *A. thaliana* GT8 protein sequences via TBtools-II’s “Blast Compare Two Seqs” identified 52 GT8 family members. An E-value threshold of e^-5^ was used for sequence alignment. The ProtParam tool on the Expasy database was used to assess the physicochemical characteristics of the *E. grandis* GT8 family members, including their molecular weight, isoelectric point, and number of amino acids (https://www.expasy.org/) (Artimo et al., 2012). The WoLFPSORT program was employed to predict the *EgGT8* proteins’ subcellular location (https://www.genscript.com/wolf-psort.html) (Horton et al., 2007).

### 2.2 Gene structure, conserved motif and conserved structural domains analysis

The MEME online tool (http://meme-suite.org) was used to predict the conserved motifs for all *EgGT8* gene family members (Bailey et al., 2009). The number of motifs was set to 10, while all other parameters were kept at their default settings. The structural information of the *EgGT8* genes was combined with the conserved motif analysis results. The data were visualized using the TBtools-II software, generating the structural and conserved motif analysis results for the *E. grandis* GT8 gene family. Furthermore, the Batch CDD NCI tool (https://www.ncbi.nlm.nih.gov/Structure/bwrpsb/bwrpsb.cgi) and TBtools-II were used to identify conserved gene domains within the *E. grandis* GT8 family members.

### 2.3 Gene family chromosomal distribution and synteny analysis

Chromosomal localization of the 52 *E. grandis* GT8 genes was performed using TBtools-II. The “Gene Location Visualize from GTF/GFF” tool in TBtools-II was employed to map the positions of the identified genes on the chromosomes. Subsequently, in the “Gene Density Profile” module of TBtools-II, the “Bin Size” was set to 100,000, with all other parameters kept at their default values. The gene sequence and annotation files of *E. grandis*, *A. thaliana*, and *P. alba* were imported into the “One Step MCScanX” tool in TBtools-II for synteny analysis. The syntenic relationships were visualized using the “Dual Synteny Plot.” Subsequently, “One Step MCScanX” was used to compare all genes within the *E. grandis* genome, and the results were visualized with “Advanced Circos” to display the intra-species synteny. When using the “One Step MCScanX” module, the value for CPU for BlastP was set to 2, the E-value was set to e^−10^, and the number of BlastHits was set to 5.

### 2.4 Phylogenetic and structural classification of gene family members

Gene sequence files for *Selaginella moellendorffii* and *Marchantia polymorpha* were retrieved from NCBI (https://www.ncbi.nlm.nih.gov/), and gene sequences for *P. alba* were obtained from the Chinese Academy of Forestry website (Liu et al., 2019). The homologous protein files for these species were generated using TBtools- II. Sequence alignment was performed utilizing MUSCLE in MEGA11.0 (Tamura et al., 2021), followed by phylogenetic tree construction using the Neighbor-Joining algorithm (NJ), with other parameters left at their default values and the bootstrap mechanism set to 1000 iterations. The resulting phylogenetic tree file was saved and structural classification was conducted using the iTOL tool (https://itol.embl.de/) (Letunic and Bork, 2024).

### 2.5 Cis-acting element analysis of gene family members

The 2000 bp sequence upstream of each *EgGT8* genes was extracted from the *E. grandis* genome data as the promoter region. The online tool PlantCARE (http://bioinformatics.psb.ugent.be/webtools/plantcare/) (Lescot et al., 2002) was used to predict the cis-acting elements present in these promoter regions. The results were visualized using TBtools-II.

### 2.6 Expression pattern analysis of gene family members in different tissues and under various stress conditions

To investigate the expression patterns of GT8 family genes across different tissues, we analyzed transcriptome data from our previous study. The plant materials and expression data of *E. grandis* GT8 family genes were obtained from the published article (Fan et al., 2024). Sampling and treatment times were conducted according to previously published literature (Jiang and Deyholos, 2006; Wang et al., 2021). The corresponding raw sequence data have been deposited in the Genome Sequence Archive (GSA) at the National Genomics Data Center under accession number PRJCA002468. For each biological replicate, we sampled at least three individual plants and performed three technical replicates per data point. Tissue separation was achieved through manual dissection: fresh stem segments were longitudinally sectioned to expose the epidermis, followed by careful removal of the outer phloem layer using fine forceps. The inner xylem tissue was subsequently collected by precision scraping. Expression patterns were visualized using TBtools-II (v1.120), where log2-transformed data underwent hierarchical clustering (Euclidean distance, complete linkage) to generate comparative heatmaps across tissue types and stress conditions. This transformation enhanced the visualization of differential expression patterns.

### 2.7 3D Structure analysis of *E. grandis* GT8 family members

The 3D structures of *EgGT8* family proteins were modeled using the SWISS-MODEL online tool (https://swissmodel.expasy.org/) through homology modeling (Waterhouse et al., 2018).

## 3 Results

### 3.1 Identification of GT8 gene family members and physicochemical property analysis in *E. grandis*

Through bioinformatics research, 52 GT8 gene family members were identified in the genome of *E. grandis* (**Table 1**). The *E. grandis* GT8 gene family members were named based on their subfamily classification and phylogenetic relationships to *A. thaliana* GT8 genes. The nomenclature of the GUX subfamily was based on previously published literature (Li et al., 2024). Physicochemical characterization of the proteins demonstrated that *EgGT8* family members exhibit amino acid lengths ranging from 134 to 1637 residues. *EgGATL4D* had the highest number of amino acids (1637), while *EgGATL4C* had the fewest (134). The average molecular weight of the proteins was 49.08 kD, with a range of 15.75 to 185.00 kD. With an average pI of 7.0, the isoelectric points varied from 4.89 to 9.51. Among them, 22 proteins (42.31%) had a pI greater than 7. The average instability index was 41.63, and members of this gene family are relatively unstable, as seen by the 21 proteins (40.38%) with an instability score below 40. The protein indices ranged from 71.68 to 100.2, with an average of 85.53. Of these, 49 proteins (94.23%) showed an average hydrophilicity coefficient of less than 0, showing that majority of the proteins in this gene family are hydrophilic (**Table 1**). According to the protein localization prediction, the GT8 family members are mainly distributed in the endoplasmic reticulum and the Golgi apparatus (**Table 1**). *A. thaliana* possesses five pairs of chromosomes, while *E. grandis* has eleven. Moreover, the GT8 gene family in *E. grandis* (52 genes) is more numerous than in *A. thaliana* (41 genes), suggesting that *E. grandis* may have undergone whole-genome expansion during evolution. This expansion likely contributed to enhanced environmental adaptability through the retention of gene duplicates and subsequent functional divergence.

**Table 1:** The basic information of identified *E. grandis* GT8 genes family members.

Rename	ID name	Number of amino acids	Molecular weight	Theoretical pI	Instability index	Aliphatic index	Grand average of hydropathicity (GRAVY)	Subcellular localization
EgGAUT1	>Eucgr.E01335.1.v2.0	675	77.16	9.19	39.41	81.05	-0.485	E.R.、Golg
EgGAUT4	>Eucgr.D00963.2.v2.0	659	75.42	9.33	39.43	84.58	-0.545	Golg
EgGAUT6	>Eucgr.B01494.1.v2.0	581	67.2	8.96	46.08	82.62	-0.553	Golg
EgGAUT7	>Eucgr.A00881.1.v2.0	636	71.32	8.64	48.77	91.78	-0.28	E.R.、Golg
EgGAUT8	>Eucgr.K02133.1.v2.0	555	63.58	9.24	33.43	85.98	-0.337	E.R.、Golg
EgGAUT9	>Eucgr.I02091.1.v2.0	554	63.21	8.31	34.09	86.41	-0.299	E.R.、Golg
EgGAUT10	>Eucgr.B00636.1.v2.0	522	59.97	6.86	45.36	91.93	-0.228	E.R.、Golg
EgGAUT12	>Eucgr.F00995.1.v2.0	533	60.84	9.08	45.05	97.69	-0.097	Golg
EgGAUT13A	>Eucgr.I01979.1.v2.0	353	61.26	8.53	41.53	94.77	-0.11	Golg
EgGAUT13B	>Eucgr.J01378.1.v2.0	533	61.02	8.79	44.91	95.48	-0.149	Golg
EgGAUT15	>Eucgr.A01962.1.v2.0	554	61.47	8.58	41.2	92.45	-0.143	E.R.、Golg
EgGATL1A	>Eucgr.H01923.1.v2.0	353	39.11	6.74	40.14	94.93	0.101	E.R.
EgGATL1B	>Eucgr.F01531.1.v2.0	426	45.84	8.77	65.24	85.73	-0.121	Nucl
EgGATL2	>Eucgr.A00485.1.v2.0	363	40.43	5.99	43.27	93.33	0.016	E.R.
EgGATL3	>Eucgr.B03054.1.v2.0	355	40.55	8.83	58.63	87.38	-0.229	E.R.、lysosome
EgGATL4A	>Eucgr.K03408.1.v2.0	356	40.32	8.4	46.33	89.55	-0.182	E.R.
EgGATL4B	>Eucgr.H01534.1.v2.0	344	39.11	9.51	41.39	88.4	-0.121	E.R.
EgGATL4C	>Eucgr.L02297.1.v2.0	134	15.75	6.82	79.75	85	-0.458	Cyto
EgGATL4D	>Eucgr.I02739.1.v2.0	1637	18.5	5.48	42.67	96.03	-0.21	E.R.
EgGATL8	>Eucgr.B02574.1.v2.0	388	43.43	8.5	58.63	83.92	-0.109	E.R.
EgGATL9	>Eucgr.I01882.1.v2.0	353	39.58	6.83	55.89	90.59	-0.081	E.R.
EgGolS1	>Eucgr.E02024.1.v2.0	365	40.71	6.15	48.57	92.71	-0.144	Golg
EgGolS2	>Eucgr.H01580.1.v2.0	319	37.22	6.41	38.15	75.83	-0.513	Cyto
EgGolS4	>Eucgr.L01806.1.v2.0	164	19.62	5.95	55.97	79.57	-0.493	E.R.
EgGolS5	>Eucgr.L03244.1.v2.0	192	22.7	5.14	24.97	72.66	-0.539	Cyto
EgGolS7	>Eucgr.H00902.1.v2.0	322	37.01	8.79	36.67	71.68	-0.419	Cyto
EgPGSIP7	>Eucgr.D02078.1.v2.0	397	45.32	8.89	32.23	100.2	0.178	E.R.、Golg
EgPGSIP8	>Eucgr.H04216.1.v2.0	395	44.1	9.25	36.17	97.47	-0.009	E.R.
EgGUX01A	>Eucgr.F04263.1.v2.0	396	46.15	6.02	36.47	79.39	-0.473	E.R.
EgGUX01	>Eucgr.L01540.1.v2.0	482	55.74	9.49	40.22	78.86	-0.428	E.R.
EgGUX02	>Eucgr.F00232.1.v2.0	600	69.75	8.92	60.2	86.92	-0.391	Golg
EgGUX03	>Eucgr.H04942.1.v2.0	639	73.74	8.07	42.14	89.45	-0.341	E.R.、Golg
EgGUX04	>Eucgr.F02737.1.v2.0	645	74.91	7.6	41.35	85.3	-0.398	E.R.、Golg
EgGUX05	>Eucgr.E04362.1.v2.0	365	40.75	6.32	47.47	92.71	-0.153	Golg
EgGUX06	>Eucgr.B01793.1.v2.0	332	38.28	5.64	30.85	83.1	-0.324	Cyto
EgGUX07	>Eucgr.B01791.1.v2.0	332	38.37	6.12	30.34	84.28	-0.323	Cyto
EgGUX08	>Eucgr.L00234.1.v2.0	332	38.45	5.64	29.03	83.98	-0.296	Cyto
EgGUX09	>Eucgr.B03987.1.v2.0	337	38.22	4.93	43.28	79.85	-0.2	Cyto
EgGUX10	>Eucgr.H02584.1.v2.0	340	38.51	5.06	43.07	81.44	-0.161	Cyto
EgGUX11	>Eucgr.H03312.1.v2.0	339	38.61	5.34	46.82	74.54	-0.397	Cyto
EgGUX12	>Eucgr.L00235.1.v2.0	335	38.28	4.93	43.02	79.43	-0.348	Cyto
EgGUX13	>Eucgr.H00906.1.v2.0	337	38.34	5.07	42.72	82.79	-0.152	Cyto
EgGUX14	>Eucgr.K03563.1.v2.0	337	38.41	4.89	41.62	86.47	-0.177	Cyto
EgGUX15	>Eucgr.L00245.1.v2.0	334	38.52	5.07	30.26	80.27	-0.276	Cyto、E.R.
EgGUX16	>Eucgr.L00251.1.v2.0	338	38.95	5.45	31.96	82.81	-0.288	Cyto
EgGUX17	>Eucgr.L00243.1.v2.0	337	38.71	5.23	32.79	81.07	-0.267	Cyto、E.R.
EgGUX18	>Eucgr.L00240.1.v2.0	337	38.78	5.23	31.22	83.92	-0.23	Cyto、E.R.、Golg
EgGUX19	>Eucgr.L00248.1.v2.0	337	38.59	5.24	27.89	80.74	-0.266	Cyto
EgGUX20	>Eucgr.L00249.1.v2.0	337	38.66	5.45	27.4	81.9	-0.261	Cyto
EgGUX21	>Eucgr.L00241.1.v2.0	338	38.81	5.39	31.05	81.69	-0.228	Cyto
EgGUX22	>Eucgr.L00250.1.v2.0	338	38.88	5.54	30.36	84.53	-0.249	Cyto
EgGUX23	>Eucgr.L01804.1.v2.0	320	37.3	5.7	39.17	72.16	-0.431	Cyto

E.R. stands for the endoplasmic reticulum, Golg refers to the Golgi apparatus, Cyto represents the cytoplasm, and Nucl denotes the nucleus.

### 3.2 The GT8 gene family members’ gene structure and conserved domains in *E. grandis*

**Figure 1:**
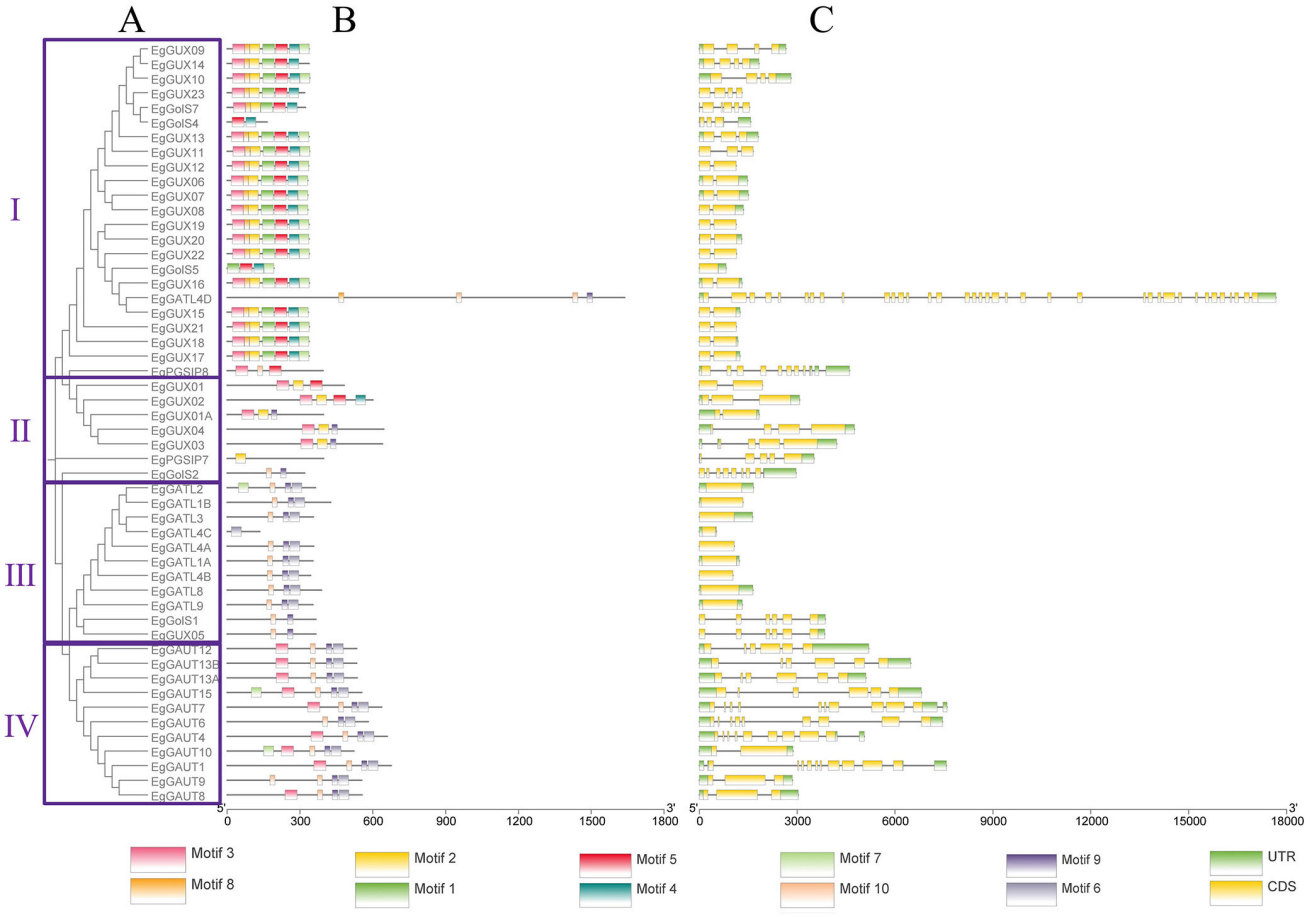
Analysis of *EgGT8* family members' gene structure and conserved motif. **(A)** Phylogenetic tree of *EgGT8* proteins using maximum likelihood methods, showing subfamily classifications. **(B)** Motif composition of *EgGT8* proteins, represented in different colors for motifs 1 through 10. **(C)** Gene models of *EgGT8*, showing the positions of exons and introns in the gene structure. The relative positions are displayed based on the kilobase scale at the bottom of the figure.

Using the *E. grandis* gene annotation file, the gene structures of the *EgGT8* family members were plotted (**Figure 1**). Ten motifs were identified in the *EgGT8* family members, and based on the conserved protein motifs, four groups were created from the phylogenetic tree of the 52 *EgGT8* members (**Figure 1A**). The domain architecture of Class I *EgGT8* family members is relatively conserved, while Classes III and IV exhibit similar domain compositions. The conserved motif analysis (**Figure 1B**) showed that all gene members contained 1 to 7 motifs, with motif 3 being the most widespread, appearing in 34 family members (65.38%). It was evident that the C-terminus of the 52 GT8 genes typically contained relatively conserved domains. *EgPGSIP7* and *EgGATL4C* only contained one conserved motif, suggesting that these genes may be incomplete or functionally impaired. Furthermore, there were differences in length, exon count, non-coding regions, and initiation sites among the different *EgGT8* genes (**Figure 1C**). The conserved domains of the *E. grandis* GT8 family members were further analyzed with the aid of Batch CDD NCI (https://www.ncbi.nlm.nih.gov/Structure/bwrpsb/bwrpsb.cgi) and TBtools-II (**Supplementary Figure 1**).

### 3.3 Chromosomal distribution of the *E. grandis* GT8 gene family

**Figure 2:**
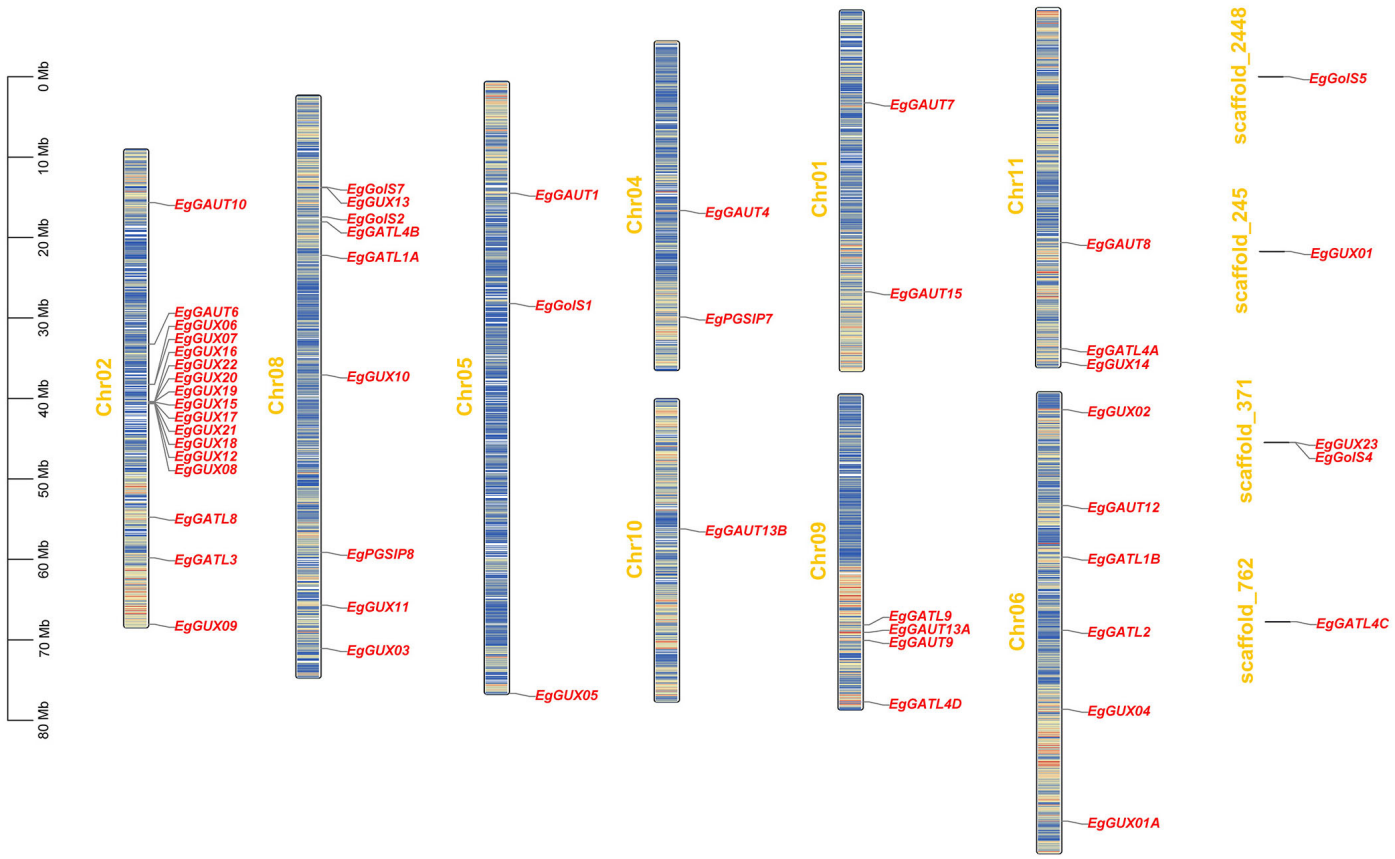
Chromosomal localization of the *E. grandis* GT8 gene family. The scale bar on the far left of the figure represents the length in megabases (Mb). The central long bars depict the chromosomes, where red regions show areas of high gene density, while blue regions show areas of low gene density. The short lines on the right side represent sequences of unknown location that have not yet been integrated into the chromosomes. The yellow text on the left of each chromosome indicates the chromosome number, while the red text on the right denotes the gene names. Each gene name is linked by a line to its specific position on the chromosome.

Tandem duplication accounts for a sizable fraction of the genes in the genome of *E. grandis* (Myburg et al., 2014). The chromosomal distribution of *EgGT8* genes was displayed using the gene annotation dataset of the *E. grandis* genome (**Figure 2**). It was discovered that there were no *EgGT8* family genes on chromosomes 3 and 7, while the remaining nine chromosomes (chr01-chr02, chr04-chr06, chr08-chr11) harbored *EgGT8* genes. Chromosomes 2 and 8 had the highest number of *EgGT8* genes, while chromosome 10 had the fewest, with only one *EgGT8* gene. On chromosome 2, *GUX* genes within the GT8 family appear in clusters. Such clustered gene arrangements might be the result of gene duplication events originating from a common ancestral gene, leading to multiple copies within the genome. The uneven distribution of GT8 family genes on chromosomes may be relevant to the replication and recombination of *GT8* genes during the evolutionary process of *E. grandis*.

### 3.4 Analysis of the GT8 gene family’s synteny within and across species in *E. grandis*

**Figure 3:**
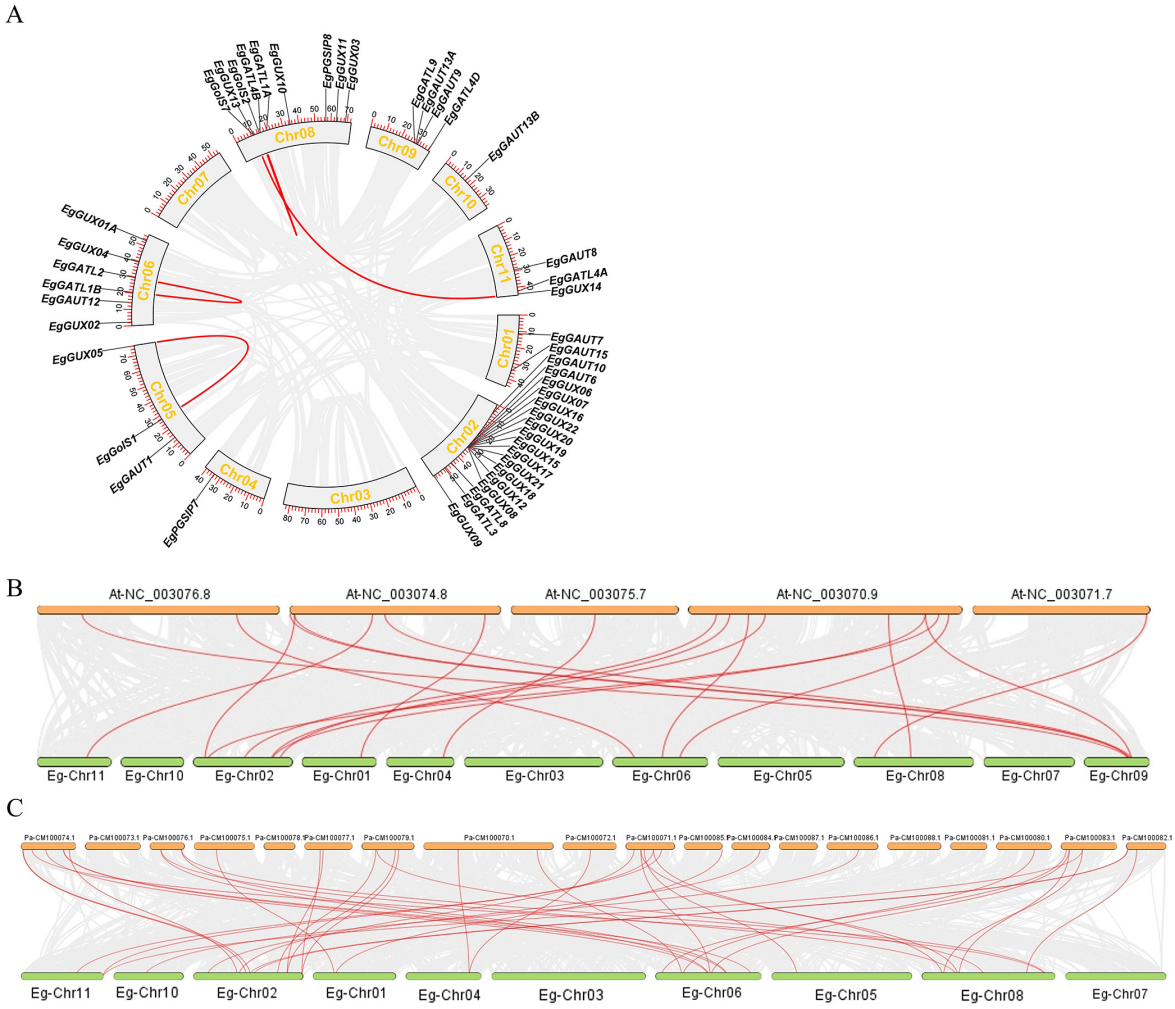
Synteny relationships of the GT8 gene family between *E*. *grandis*, *A*. *thaliana*, and *P. alba.* **(A)** Intra-species synteny relationships of the *E*. *grandis* GT8 gene family, with gray lines representing all syntenic blocks in the GT8 genome and red lines indicating segmental duplications of the *EgGT8* genes. **(B)** Inter-species synteny between *E*. *grandis* and *A*. *thaliana*, and **(C)** between *E*. *grandis* and *P. alba*, with gray lines representing all syntenic blocks and red lines indicating synteny of GT8 genes between species.

Three main evolutionary forces are thought to be tandem gene duplication, segmental duplication, and whole-genome duplication (Panchy et al., 2016). To explore the whole-genome duplication of *EgGT8* genes, an intra-species synteny analysis of the *E. grandis* GT8 gene family was conducted (**Figure 3A**). The finding showed that the GT8 gene family in *E. grandis* underwent relatively low-frequency self-duplication, with only four pairs of tandem and segmental duplications: *EgGATL4B* and *EgGolS2, EgGUX13* and *EgGUX14, EgGATL2* and *EgGATL1B*, *EgGUX05* and *EgGolS1*.

Further inter-species synteny analysis using the genomes of *A. thaliana* and *P. alba* revealed 19 syntenic pairs between *A. thaliana* and *E. grandis* (**Figure 3B**), and 39 syntenic pairs between *P. alba* and *E. grandis* (**Figure 3C**). This suggests a closer phylogenetic relationship between *P. alba* and *E. grandis*.

### 3.5 Phylogenetic tree of the *E. grandis* GT8 gene family

**Figure 4:**
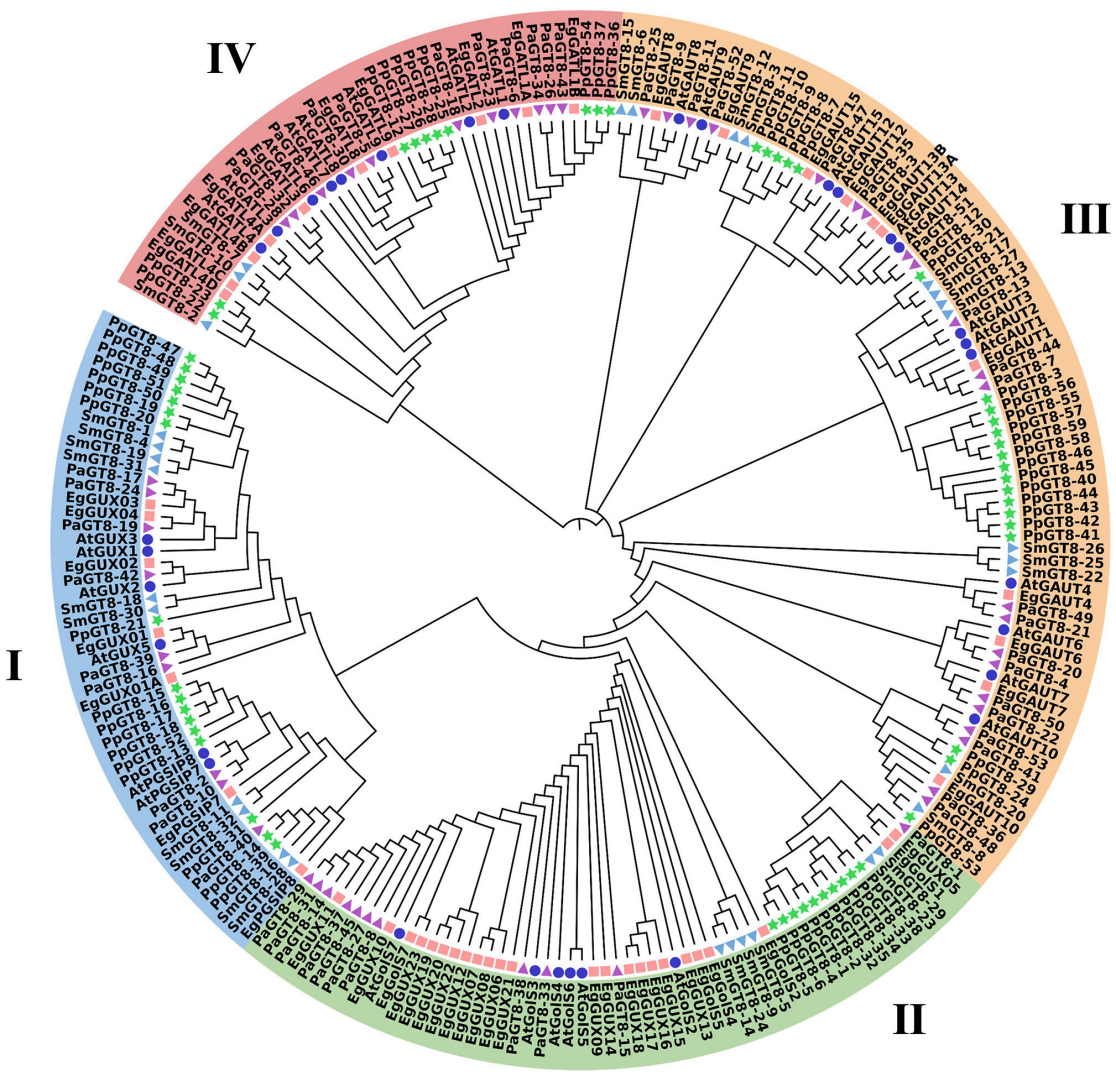
Phylogenetic tree of GT8 gene family members. The GT8 genes from *E. grandis* (52), *A. thaliana* (33), *S. moellendorffii* (32), *P. patens* (59), and *P. alba* (53) were selected. These species are represented by pink squares, blue circles, light blue triangles, green stars, and purple inverted triangles, respectively. MEGA11 was used to create the maximum-likelihood phylogenetic tree using full-length GT8 sequences (5000 bootstrap replicates). Blue, green, orange, and pink highlight the four groups.

MEGA software was used to evaluate 229 protein sequences from *E. grandis* (52), *A. thaliana* (33), *S. moellendorffii* (32), *P. patens* (59), and *P. alba* (53) to comprehend the evolutionary connections of the *E. grandis* GT8 family members with other species. The phylogenetic tree analysis divided the GT8 proteins into four groups (Group I–IV) (**Figure 4**). The *EgGT8* family had 7, 24, 11, and 10 members in Groups I–IV, respectively. Based on the phylogenetic relationships, it was found that *EgGUX02* and *EgGUX04* displayed closer links with *AtGUX1* and *AtGUX2*; *EgGolS4* and *EgGolS5* showed closer relationships with *AtGolS2* and *AtGolS4*; *EgGAUT1* and *EgGAUT12* were more closely related to *AtGAUT1/PARVUS* and *AtGAUT12; EgGATL8* and *EgGATL4A* showed closer relationships with *AtGATL4* and *AtGATL9*. Phylogenetic analysis revealed that *EgGT8* family members clustered closely with orthologs from *A. thaliana* and *P. alba*, forming a distinct clade separate from the more evolutionarily distant species *S. moellendorffii* and *P. patens*.

### 3.6 Analysis of cis-acting elements in the promoters of the *E. grandis* GT8 gene family

**Figure 5:**
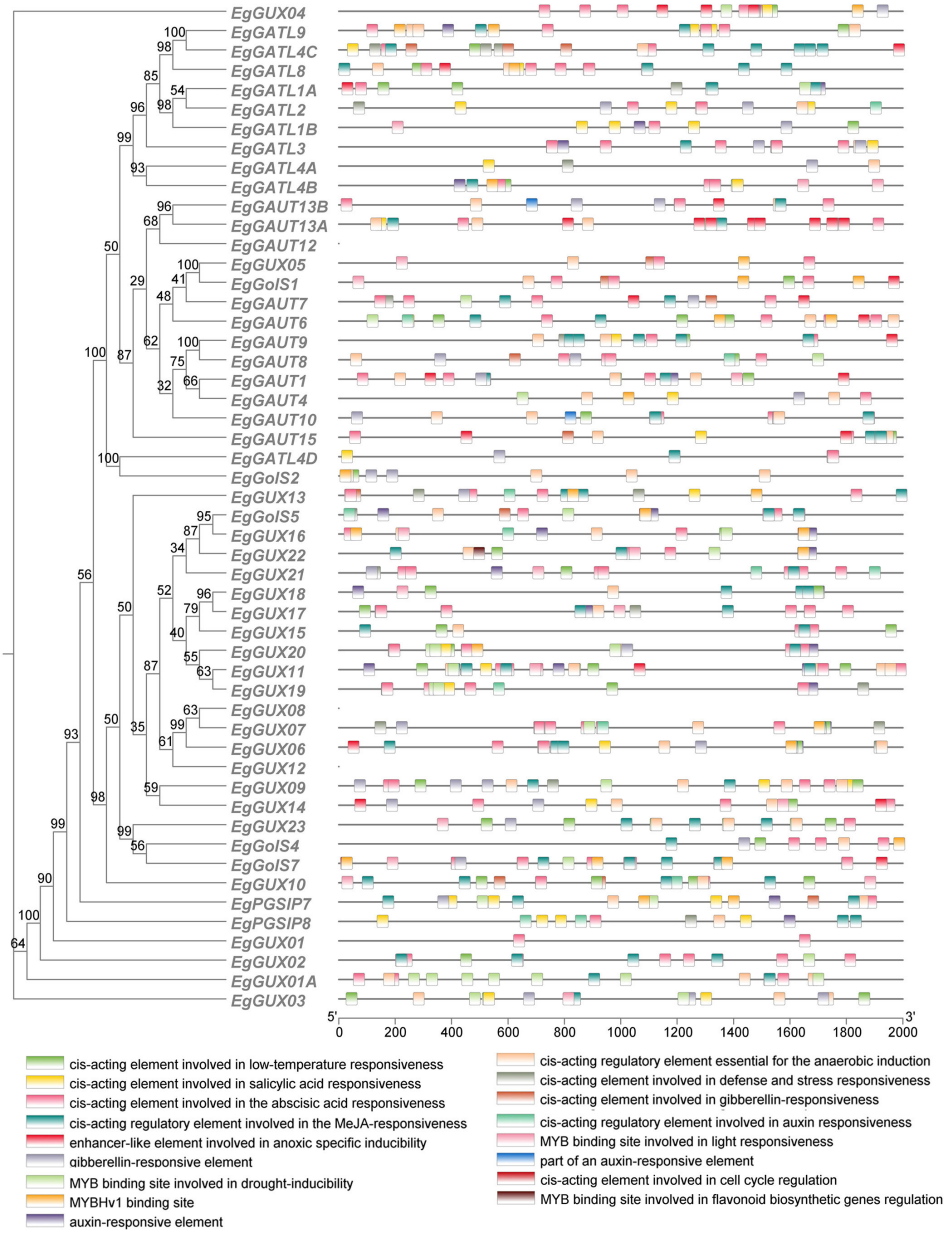
Cis-acting element analysis of the *E. grandis* GT8 gene family promoters. The vertical axis of the figure represents the names of different genes, while the horizontal axis represents the 2000 bp upstream region of the gene. The squares in the middle indicate the positions of various cis-acting elements, with their specific names shown in the upper right corner.

By binding to cis-acting sites in the promoter regions, transcription factors (TFs) can regulate the expression of genes (Wang et al., 2014). We analyzed cis-acting elements within the 2000 bp promoter regions upstream of *EgGT8* genes and correlated these findings with phylogenetic relationships (**Figure 5**). Among the 52 *EgGT8* family genes, 17 cis-acting elements were predicted. These elements were classified into several categories: hormone response elements, light response elements, abiotic stress response elements, and plant growth and development-related elements. Light response-related elements were the most prevalent, and hormone response elements such as salicylic acid (SA), abscisic acid (ABA), methyl jasmonate (MeJA), gibberellin (GA), and auxin (IAA) response elements were present in all family members. This suggests that light and plant hormone signaling may control the transcription of this gene family. Additionally, there were four types of abiotic stress response elements and two plant growth-related elements.

### 3.7 Expression of the *E. grandis* GT8 gene family in different tissues

**Figure 6:**
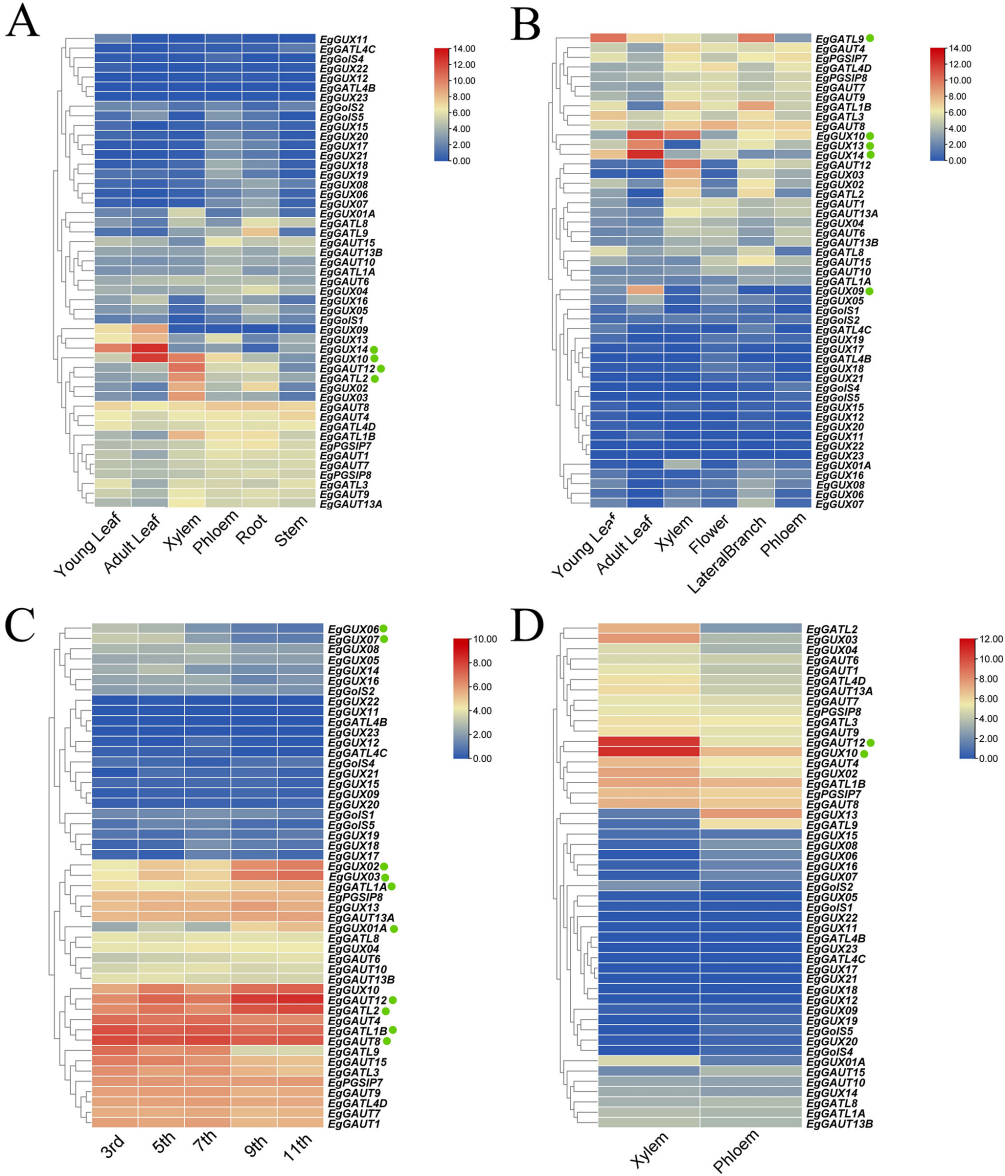
Heatmap of *EgGT8* Gene Expression in Various *E*. *grandis* Tissues. **(A)** Expression heatmap of 6-month-old *E*. *grandis* in roots, stems, xylem, phloem, juvenile leaves, and mature leaves. **(B)** 3-year-old *E. grandis* expression heatmap in phloem, xylem, flowers, lateral branches, immature leaves, and mature leaves. **(C)** *EgGT8* gene expression heatmap in 6-month-old *E*. *grandis* stem nodes (nodes 3, 5, 7, 9, 11). **(D)** *EgGT8* gene expression heatmap in 6-year-old *E*. *grandis* xylem and phloem. The color of the rectangular grids represents expression levels, with red indicating higher expression and blue indicating lower expression. The green dots next to the genes indicate key genes of interest.

To investigate the expression patterns of the *EgGT8* gene family in *E. grandis*, we analyzed 49 *EgGT8* genes across multiple tissues, including young leaves, mature leaves, phloem, xylem, roots, flowers, stems, and lateral branches. Expression data were unavailable for *EgGolS7*, *EgGATL4A* and *EgGUX15.* The results showed that in 6-month-old *E. grandis*, *EgGUX14* was highly expressed in young leaves and mature leaves, while *EgGUX10* exhibited significant expression in mature leaves and xylem. Compared to other tissues, *EgGAUT12* and *EgGATL2* showed relatively higher expression levels in the xylem (**Figure 6A**). The expression of the *EgGT8* genes in various sections of 3-year-old *E. grandis* is shown in **Figure 6B**. *EgGATL9* was most expressed in young leaves and lateral branches, whereas *EgGUX09*, *EgGUX13*, *EgGUX14*, and *EgGUX10* were most expressed in mature leaves. **Figure 6C** demonstrates the expression of the *EgGT8* genes in various stem segments of 6-month-old *E. grandis*, with *EgGAUT12*, *EgGAUT8*, *EgGATL2* and *EgGATL1B* exhibiting the highest expression. From the top to the base of the tree, the expression of *EgGUX02*, *EgGUX03*, *EgGUX01A* and *EgGATL1A* gradually increased, while the expression of *EgGUX06*, *EgGUX07* and *EgGATL9* gradually decreased. In comparison, the expression levels in the xylem were higher than those in the phloem, with *EgGAUT12* and *EgGUX10* showing the highest expression in the xylem (**Figure 6D**).

### 3.8 Expression of *E. grandis* GT8 family members under abiotic stress and plant hormone treatments

**Figure 7:**
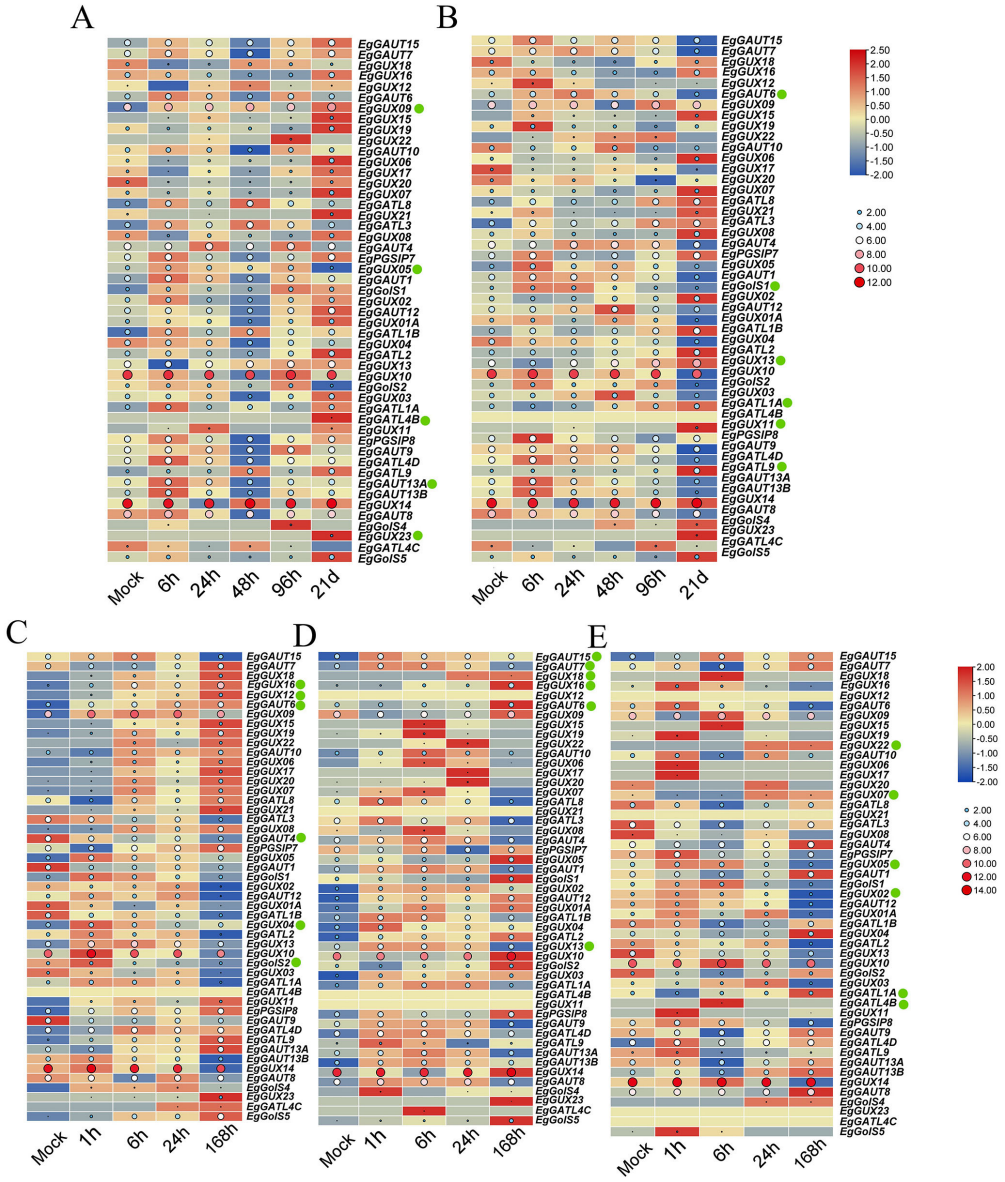
Heatmap of *EgGT8* Gene Expression in *E*. *grandis* under abiotic stress and plant hormone treatments. **(A)** Expression heatmap after phosphate deficiency treatment (0, 6, 24, 48, 96 hours, 21 days). **(B)** Expression heatmap after boric acid deficiency treatment (0, 6, 24, 48, 96 h, 21 days). **(C)** Expression heatmap after salt stress treatment (0, 1, 6, 24, 168 h). **(D)** Expression heatmap after MeJA treatment (0, 1, 6, 24, 168 h). **(E)** Expression heatmap after SA treatment (0, 1, 6, 24, 168 h). The color of the rectangular grids represents expression levels, with red indicating higher expression and blue indicating lower expression. The size and color of the circles correspond to the magnitude of the raw data: larger, redder circles indicate higher expression levels, while smaller, bluer circles indicate lower expression levels. The green dots next to the genes indicate key genes of interest.

To investigate the differential expression of *EgGT8* family members under abiotic stress and plant hormone treatments, 2-month-old *E. grandis* seedlings were subjected to phosphate deficiency, boric acid deficiency, and salt stress. Additionally, salicylic acid (SA) and methyl jasmonate (MeJA) were sprayed on the leaves. Expression analysis was performed on 49 *EgGT8* genes, excluding *EgGolS7, EgGATL4A*, and *EgGUX15*. The circles in the figure provide a more intuitive visualization of gene expression levels, with larger circles indicating higher expression. This design facilitates vertical comparisons of gene expression across different treatments. The normalized heatmaps indicated that under phosphate deficiency, the expression of *EgGATL4B, EgGUX09*, and *EgGUX23* gradually increased, while the expression of *EgGUX05* and *EgGAUT13A* gradually decreased (**Figure 7A**). Under boric acid deficiency, the expression of *EgGUX11, EgGUX13*, and *EgGATL1A* gradually increased, while the expression of *EgGATL9, EgGAUT6*, and *EgGolS1* gradually decreased (**Figure 7B**). Under salt stress, the expression of *EgGUX12, EgGUX16*, and *EgGAUT6* gradually increased, while the expression of *EgGAUT4, EgGUX04*, and *EgGolS2* gradually decreased (**Figure 7C**). Under MeJA treatment, the expression of *EgGUX16, EgGUX18*, and *EgGAUT6* gradually increased, while the expression of *EgGAUT7, EgGAUT15*, and *EgGUX13* gradually decreased (**Figure 7D**). Under SA treatment, the expression of *EgGUX07, EgGUX22*, and *EgGATL1A* gradually increased, while the expression of *EgGUX02, EgGUX05*, and *EgGATL4B* gradually decreased (**Figure 7E**). It is worth noting that the increases or decreases in expression observed here may reflect short-term effects under different treatments, which might be regulated back due to feedback mechanisms after prolonged treatments.

### 3.9 3D structure analysis of *E. grandis* GT8 gene family members

**Figure 8:**
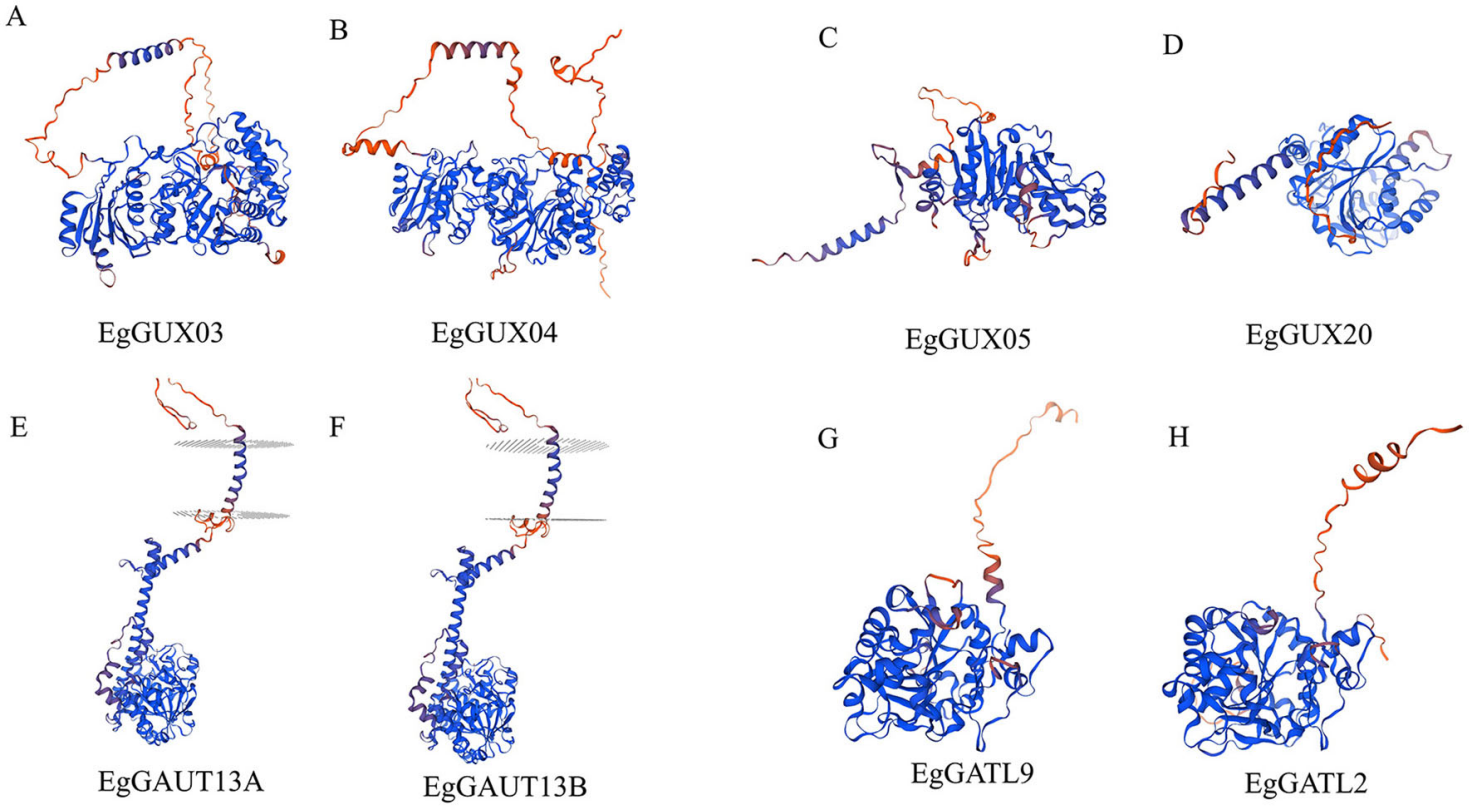
3D Structures of *EgGT8* Gene Family Members. **(A)** Protein subfamily I's three-dimensional structure *EgPGSIP7*. **(B)** Protein subfamily I's three-dimensional structure *EgPGSIP8.* **(C)** 3 Protein subfamily II's three-dimensional structure *EgGUX05.* **(D)** Protein subfamily II's three-dimensional structure *EgGUX20.* **(E)** 3 Protein subfamily III's three-dimensional structure *EgGAUT13A.* **(F)** Protein subfamily III's three-dimensional structure *EgGAUT13B.* **(G)** Protein subfamily IV's three-dimensional structure *EgGATL9.* **(H)** Protein subfamily IV's three-dimensional structure *EgGATL2.*.

Using SWISS-MODEL, the 3D structures of eight sample *EgGT8* proteins were effectively predicted (**Figure 8**). Proteins within the same subfamily showed significant similarity in their 3D structures with minimal differences. However, proteins from different subfamilies exhibited more noticeable structural differences, which may be attributed to variations in the α-helix, β-turn, and irregular coil regions. These structural differences likely result in changes in the spatial folding angles, which may underpin the different functional roles of these proteins.

## 4 Discussion

The GT8 gene family is widely present in plant genomes, and studies have demonstrated that it is essential for plant cell wall biosynthesis and reaction to abiotic stress. *E. grandis*, characterized by its rapid growth and high economic value, is a key species for wood reserves. Therefore, studying the functional differentiation and evolution of the GT8 gene family in *E. grandis* is vital for the formation and utilization of its wood. Currently, the GT8 family has been reported in various species, but research on the GT8 family in woody plants such as *E. grandis* is relatively scarce. Using the GT8 genes of *A. thaliana* as a reference, we discovered 52 GT8 genes in *E. grandis*. Analysis of their physicochemical properties showed that the *EgGT8* genes are unevenly distributed across 11 chromosomes, with most family members being unstable. Subcellular localization predictions displayed that *EgGT8* proteins are extensively dispersed in the Golgi apparatus and endoplasmic reticulum, and the bulk of these genes (94.23%) encode hydrophilic proteins. The *E. grandis* GT8 family’s protein sequences and domains are generally very conserved, indicating that these genes are important for plant physiology, warranting further functional exploration.

Ten GT8 genes (*EgGUX08*, *EgGUX12*, *EgGUX15*, *EgGUX16*, *EgGUX17*, *EgGUX18*, *EgGUX19*, *EgGUX20*, *EgGUX21*, and *EgGUX22*) form a tightly linked cluster on *E. grandis* chromosome 2 (**Figure 2**), showing high structural similarity-a hallmark of tandem duplication. These group II genes share conserved motifs and domains, suggesting retained biochemical functions and potential involvement in common pathways. Analysis of *E. grandis* GT8 genes identified only four collinear gene pairs: *EgGATL4B* and *EgGolS2, EgGUX13* and *EgGUX14, EgGATL2* and *EgGATL1B*, *EgGUX05* and *EgGolS1* (**Figure 3A**). These genes represent non-contiguously distributed repetitive sequences in the genome, with phylogenetic evidence supporting their origin through dispersed duplication events. Syntenic gene pairs exhibit conserved motifs and similar gene architectures (**Figure 1**), indicating their potential functional conservation via dosage effects. In contrast, non-syntenic gene pairs demonstrate substantial divergence in gene structures, suggesting evolutionary functional differentiation. Such divergent paralogs likely mediate distinct biological processes contributing to growth and development in *E. grandis*.

Comparative genomic analysis revealed that *E. grandis* exhibits significantly stronger collinearity in GT8 genes with *P. alba* than with *A. thaliana* (**Figures 3B, C**). This phenomenon can be attributed to two primary factors: First, both *E. grandis* and *P. alba* belong to woody clade, while *A. thaliana* is classified to herbaceous clade, indicating closer phylogenetic relationship between the former two species. Second, as woody plants, *Eucalyptus* and *Populus* require more conserved GT8 gene functions for secondary cell wall formation, resulting in greater gene diversification.

*E. grandis* exhibits greater GT8 gene family expansion (11 chromosome pairs) compared to *A. thaliana* (5 pairs) (**Figure 7B**), likely due to whole-genome duplication. This expansion facilitated functional diversification of GT8 genes, enhancing environmental adaptation. Duplicated genes may evolve specialized functions through sequence divergence and expression changes, forming adaptive networks. Future studies could employ CRISPR and comparative genomics to investigate GT8 functional mechanisms.

Previous work has demonstrated that *IRX8*, a member of the GT8 gene family, contributes to the production of the tetrasaccharide that contains galacturonic acid at the reducing end of xylan (Brown et al., 2007). The protein encoded by *GAUT1* functions as a galacturonosyltransferase, directly participating in pectin biosynthesis (Caffall and Mohnen, 2009). In the phylogenetic tree constructed for GT8 genes across *E. grandis*, *A. thaliana*, *S. moellendorffii*, *P. patens*, and *P. alba*, these genes cluster within Group III. This suggests that *EgGAUT1*, *EgGAUT12*, and other Group III members may share functional similarities with *IRX8* and *GAUT1*, potentially contributing to both pectin and xylan biosynthesis. Additionally, *EgGUX02* and *EgGUX04* exhibit close phylogenetic relationships with *AtGUX1* and *AtGUX2*, indicating their possible involvement in xylan side-chain modifications by incorporating glucuronic acid (GlcA) into the xylan backbone (Rennie et al., 2012; Lyczakowski et al., 2021). Similarly, *EgGolS4* and *EgGolS5* cluster with *AtGolS2* and *AtGolS4*, suggesting their potential roles as essential enzymes in the production of raffinose family oligosaccharides (Taji et al., 2002). Furthermore, *EgGATL8* and *EgGATL4A* are closely related to *AtGATL4* and *AtGATL9*, suggesting their possible role in the formation of the tetrasaccharide that contains galacturonic acid at the reducing end of xylan (Peña et al., 2007). These results offer insightful information about the functional diversification of GT8 genes, yet further experimental validation and functional characterization are required in future studies.

Expression data from *E. grandis* seedlings under various abiotic stresses and plant hormone treatments underscore the importance of MeJA. MeJA is a significant plant hormone, and previous studies have demonstrated that MeJA is crucial for maintaining ion homeostasis (Mir et al., 2018) and in cell stress responses (Piotrowska et al., 2009; Bali et al., 2019; Shri et al., 2019). In MeJA-treated *E. grandis* seedlings, the expression of GT8 genes was generally upregulated. The cis-element analysis showed that all *EgGT8* family members contain hormone response elements, including those responsive to MeJA, strongly indicating a strong bond between the *EgGT8* gene family and MeJA, which could be further analyzed in subsequent studies.

In situations including salt stress, phosphorus deficiency, boric acid deficiency, SA treatment, and MeJA treatment, *EgGUX10* and *EgGUX14* displayed increased levels of expression, according to heatmap analysis of RNA-Seq data. This suggests that these two genes may participate in hormone signal transduction pathways. As key nodes in the signaling chain, they may respond to hormone signals and regulate the expression of downstream genes, thus coordinating the plant’s response to different environmental signals. Moreover, *EgGUX10* and *EgGUX14* might function synergistically under environmental stress, controlling plant development and growth as well as adversity adaptation.

Under both boric acid deficiency and salicylic acid (SA) treatment, the expression level of the *EgGATL1A* gene was significantly upregulated, suggesting its involvement in both biotic and abiotic stress responses (**Figure 7**). Phylogenetic analysis reveals that *EgGATL1A* clusters with *AtGATL1*, which have known to mediate glucuronoxylan biosynthesis during secondary cell wall thickening, in the same evolutionary clade, demonstrating high sequence and functional similarity (Lee et al., 2007). We therefore propose that *EgGATL6* similarly participates in glucuronoxylan biosynthesis.

Tissue-specific expression analysis of the GT8 gene family in *E. grandis* reveals that several *EgGT8* members are highly expressed in xylem and phloem, while others are predominantly expressed in leaves. Overall, *EgGT8* genes exhibit highest expression levels in xylem, mature leaves, and lateral branches. This expression pattern aligns well with the enrichment of MYB-binding cis-elements in the promoter regions of GT8 genes, suggesting that these genes may be regulated by MYB transcription factors and participate in secondary cell wall modification. Based on the structural features of the GT8 gene family, we propose a hypothesis that the MYB-GT8 module enhances mechanical strength of the cell wall by glycosylating lignin precursors. This modification helps *E. grandis* respond to mechanical stress, maintain leaf morphology and structure, and regulate the synthesis and reinforcement of cell walls in lateral branches, thereby ensuring normal photosynthesis and promoting lateral branch development. This proposed mechanism is consistent with functional studies of *PtrGT8D* in *Populus* (Li et al., 2011), although tandemly duplicated genes in the *E. grandis* GT8 family may have further diverged to acquire subfunctions in regulating different substrates.

To evaluate the natural selection pressures acting on the *E. grandis* GT8 gene family, we calculated the ratio of non-synonymous (Ka) to synonymous (Ks) substitutions (u) for the *EgGT8* gene pairs (**Supplementary Table 1**). When the Ka/Ks ratio is < 1, = 1, or > 1, it indicates purifying, neutral, and positive selection, respectively. The data suggest that the majority of these gene pairs are under purifying selection, with only a few exhibiting signs of neutral selection. This likely indicates that these genes have been primarily subjected to conserved selection throughout evolution to maintain their functional integrity.

## 5 Summary

We analyzed 52 members of the GT8 gene family in *E. grandis* from the perspectives of phylogeny, gene structure, and proteomics. These genes were classified into four subfamilies, and a series of conserved protein structures were identified. We analyzed the phylogenetic relationships between different species. Expression profile analysis of related genes under different tissue types and treatments indicated that members of the GT8 gene family play important roles in plant resistance to environmental stress, a process associated with their involvement in multiple cell wall biosynthesis pathways. Based on the distribution of key GT8 family members from published literature and expression profile data, we identified potential key genes that may function within the *E. grandis* GT8 family. This will assist in future functional studies and provide valuable insights for breeders in improving wood quality and cultivating superior varieties.

## Data Availability

The datasets presented in this study can be found in online repositories. The names of the repository/repositories and accession number(s) can be found in the article/**Supplementary Material**.

## Supplementary material

Supplementary Figure 1 Conserved Domains of the *E. grandis* GT8 Gene Family This figure illustrates the conserved domains of the *E. grandis* GT8 gene family and their spatial distribution. The left side of the image displays the names of different genes, while the middle segments, scaled in kilobases, indicate the relative positions of the conserved domains. Different colored blocks represent distinct conserved domains, with their corresponding names labeled at the bottom of the image.

Supplementary Table 1 Ka/Ks Selection Pressure Analysis of *Eucalyptus grandis* GT8 Gene Family Members.
