# Supplementary figures and images for: Comprehensive genome-wide identification and functional analysis of the GT8 gene family in Eucalyptus Grandis
Source: Front Plant Sci. 2025 Jun 19;16:1610059. doi: 10.3389/fpls.2025.1610059 (PMC12222175; doi:10.3389/fpls.2025.1610059)

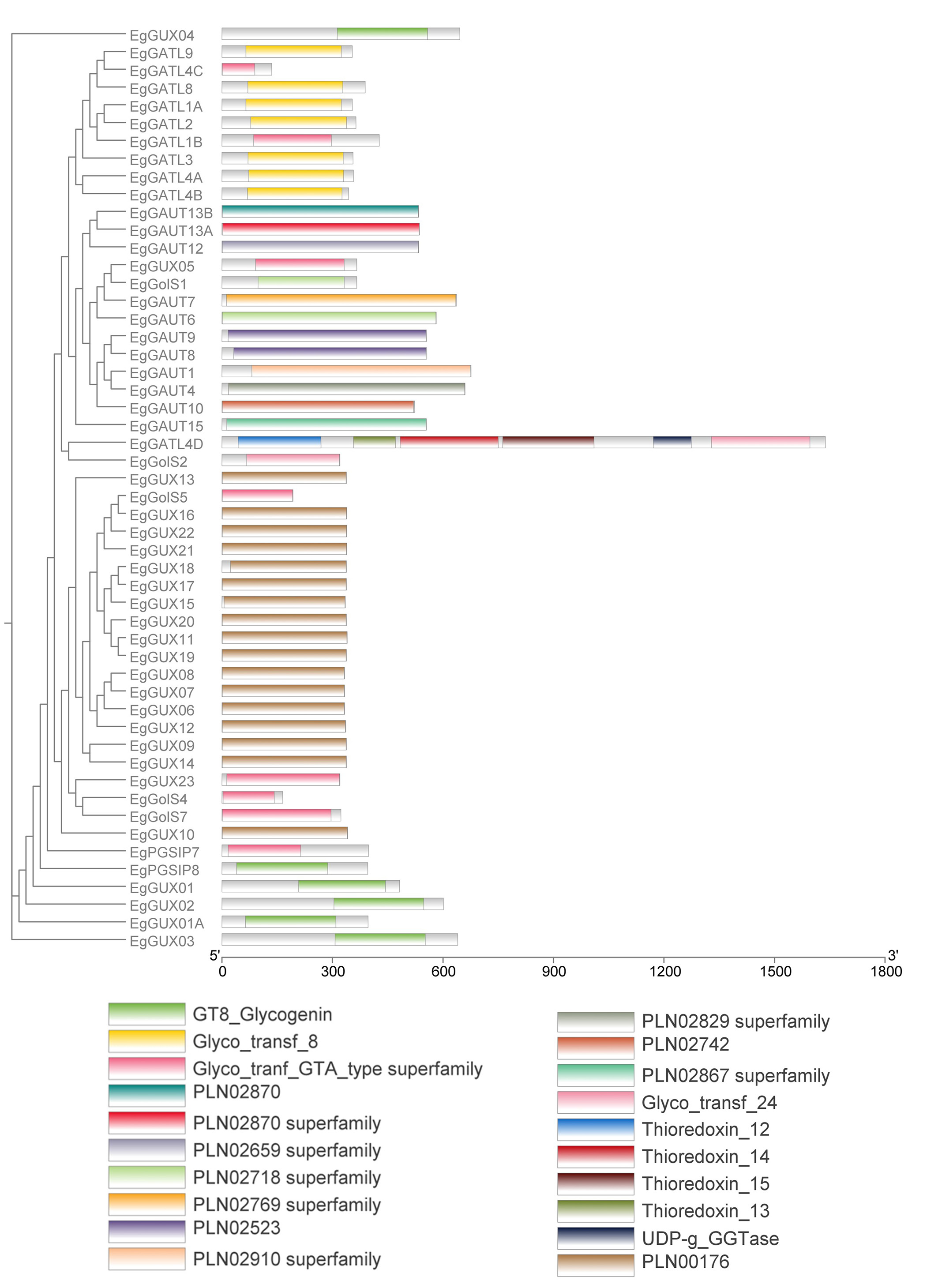

Supplement: Supplementary Figure 1 — Conserved Domains of the E. grandis GT8 Gene Family This figure illustrates the conserved domains of the E. grandis GT8 gene family and their spatial distribution. The left side of the image displays the names of different genes, while the middle segments, scaled in kilobases, indicate the relative positions of the conserved domains. Different colored blocks represent distinct conserved domains, with their corresponding names labeled at the bottom of the image. [file Image1.jpeg]
