# Supplementary material for: Comprehensive genome-wide identification and functional analysis of the GT8 gene family in Eucalyptus Grandis
Source: Front Plant Sci. 2025 Jun 19;16:1610059. doi: 10.3389/fpls.2025.1610059 (PMC12222175; doi:10.3389/fpls.2025.1610059)
Supplement: Supplementary Table 1 — Ka/Ks Selection Pressure Analysis of Eucalyptus grandis GT8 Gene Family Members. [file Table1.docx]

Supplementary Table 1. Ka/Ks Selection Pressure Analysis of *Eucalyptus grandis* GT8 Gene Family Members.

| Seq1 | Seq2 | Ka | Ks | Ka/Ks Ratio |
| --- | --- | --- | --- | --- |
| EgGolS1 | EgGUX05 | 0.002401924 | 0.02731121 | 0.087946436 |
| EgGATL1B | EgGATL2 | 0.281800387 | 0.65775664 | 0.428426516 |
| EgGolS2 | EgGATL4B | 0.635117734 | 1.18023606 | 0.538127716 |
| EgGUX13 | EgGUX14 | 0.104374818 | 0.93273916 | 0.1119014 |
